# Manufacturing of high-quality green CSA-supported OPC cement with optimum physical and mechanical properties

**DOI:** 10.1038/s41598-025-14008-w

**Published:** 2025-08-09

**Authors:** Hala A. Hossein, Bassem S. Nabawy, Samah A. Sanad, E. A. El-Alfi

**Affiliations:** 1 Refractories, Ceramics & Building Materials Department, National Research Centre, Giza, Egypt; 2 Department of Geophysical Sciences, National Research Centre, Giza, Egypt; 3 Raw Building Materials & Processing Technology Research Institute, Housing and Building National Research Centre (HBRC), Giza, Egypt

**Keywords:** Calcium sulfoaluminate green cement, Ye’elimite, Low-energy, Physical and mechanical properties, Civil engineering, Energy infrastructure, Ceramics

## Abstract

In the current study, we aim to enhance the physical and mechanical properties of the ordinary Portland cement (OPC) by incorporating small amounts of calcium sulfoaluminate (CSA) at a rate of 0, 1, 2 and 3% (mixtures A, B, C, and D) to improve its physical and mechanical properties. These mixtures allowed the CSA clinker to emit less CO_2_ and clink at a low temperature of 1250 °C, which is significantly lower than that of the OPC clinker (1450 °C). The apparent density, specific gravity, porosity, and unconfined compressive strength (UCS) of the pure OPC (0%, mixture A) and the OPC-CSA pastes (B, C, & D mixtures) were measured at curing periods of 1, 3, 7, 28, and 90 days. The scanning electron microscopy (SEM) coupled with the energy dispersive X-ray (EDS) was employed to investigate the internal structure of these pastes, while the XRF technique was employed to determine their chemical composition. Additionally, the optical characteristics of the formulated and disintegrated phases were delineated using FTIR spectroscopy, while the differential thermal analysis (DTA) and thermogravimetric analysis (TGA) were employed to examine them as the curing age increased. The mixture C (2% CSA + 98% OPC) sintered at 90 days is characterized by the best physical and mechanical properties (compressive strength = 52.5 MPa, porosity = 2.86%, apparent density = 2.015 g/cc, and specific gravity = 2.405 g/cc). This procedure is beneficial on a laboratory scale and applicable on the industrial scale to improve the physical and mechanical characteristics of the OPC and decrease its energy consumption.

## Introduction

Construction is one of the largest industrial sectors globally, and concrete plays a crucial role in this industry. Consequently, in recent decades, the growing demand for cement’s unique properties has led to the development of numerous cement types. Among these, the Ordinary Portland Cement (OPC) is the most common energy building material. The Portland cement production not only consumes giant quantities of energy and calcite but also releases undesirable amounts of CO_2_ emissions, which are about 4 billion tons annually^1–4^. The Portland cement industry, worldwide, emits more than 5% of carbon, where each ton of the ordinary Portland cement (OPC) produces more than 0.97 ton of equivalent carbon dioxide (CO_2_-e), which is a considerable fraction of greenhouse gases^5–10^. The most substantial aims in lowering the energy consumption and decreasing the CO_2_ emissions in the cement industry can be achieved during the pyroprocess by utilizing biomass and other non-traditional alternative fuel sources owing to energy with minimized near net-zero carbon-based emissions^11^.

In the last decades, calcium sulfoaluminate (CSA) cement has been developed as a type of special cement, which was first developed in the sixties^12^, providing a preliminary drawing with a more intriguing contrast to the OPC. Generally, the production of CSA cements consumes less energy during the manufacturing process, operating at a temperature range of 1200–1300 °C compared to approximately 1450 °C for OPC, resulting in an energy reduction of about 16–25%^13–15^. This savings may be attributed to the lower energy consumption and CO_2_ emissions during the production process^3^. Furthermore, these cements consume low grinding energy, resulting in downy and tenuous clinker, 40% less CO_2_ emission, saving the amounts of the utilized natural raw materials by replacing them with various industrial by-products, and presenting great impedance to sulfate attack^16–19^. Calcium sulfoaluminate (CSA), belitic sulfoaluminate, and ferrosulfoaluminate are types of eco-friendly CSA cements contingent on their application. Additionally, natural raw materials like clays, limestone, iron ores, and bauxite, as well as industrial wastes and by-products such as galvanic sludge, pyrite ash, fly ash, phosphogypsum, and metallurgical slag, can be used to make CSA cements.

The main part of the CSA clinker is called Klein’s component, which is made up of 4CaO.3AI_2_O_3_.SO_3_ (C_4_A_3_ Š) and includes ye’elimite, Ca_2_SiO_4_ (C_2_S) beIite, C4AF (brownmillerite), and sometimes calcium aluminates (C_12_A_7_ and C_11_A_7_CaF_2_). This main phase of CSA cement (ye’elimite) liberates 0.216 g CO_2_ per gram of phase, while alite (C_3_S), the major compound of the OPC, emits 0.578 g CO_2_ per gram of phase through the calcination process^20,21^. Unlike the OPC, which gets its UCS from the calcium silicate hydration process, the belite (C_2_S) and the alite (C_3_S), the CSA cements acquire their CS from the ye’elimite hydration with calcium sulfate (gypsum CŠH_2_ or anhydrite CŠ) to produce ettringite and monosulfate through these reactions^22–25^.

1 $${{\text{C}}_{\text{4}}}{{\text{A}}_{\text{3}}}\check{S}{\text{ + 8C}}\check{S}{\text{ + 6CH}}\,{\text{+}}\,{\text{90H }} \to {\text{3}}{{\text{C}}_{\text{6}}}{\text{A}}{\check{S}_{\text{3}}}{{\text{H}}_{{\text{32}}}}$$

2 $${{\text{C}}_{\text{4}}}{{\text{A}}_{\text{3}}}\check{S}{\text{ }}+{\text{ 2C}}\check{S}{\text{ }}+{\text{ 38H }} \to {\text{ }}{{\text{C}}_{\text{6}}}{\text{A}}{\check{S}_{\text{3}}}{{\text{H}}_{{\text{32}}}}+{\text{ 2A}}{{\text{H}}_{\text{3}}}$$

3 $${{\text{C}}_{\text{4}}}{{\text{A}}_{\text{3}}}\check{S}{\text{ + 18H }} \to {\text{ }}{{\text{C}}_{\text{4}}}{\text{A}}\check{S}{{\text{H}}_{{\text{12}}}}\,+\,{\text{2A}}{{\text{H}}_{\text{3}}}$$

4 $${{\text{C}}_{\text{4}}}{{\text{A}}_{\text{3}}}\check{S}{\text{ + 2C}}\check{S}{{\text{H}}_{\text{2}}}\,{\text{+}}\,{\text{34H }} \to {\text{ }}{{\text{C}}_{\text{6}}}{\text{A}}{\check{S}_{\text{3}}}{{\text{H}}_{{\text{32}}}}+{\text{ 2A}}{{\text{H}}_{\text{3}}}$$

5 $${{\text{C}}_{\text{4}}}{{\text{A}}_{\text{3}}}\check{S}{\text{ + 2C}}\check{S}{{\text{H}}_{\text{2}}}\,+\,{\text{6CH}}\,+\,{\text{26H }} \to {\text{ 3}}{{\text{C}}_{\text{4}}}{\text{A}}\check{S}{{\text{H}}_{{\text{12}}}}$$

The hydration products of C_4_A_3_Š (ye’elimite) are conditional on the sulfate and lime content. In the presence of Ca(OH)_2_ and sulfate, ye’elimite forms ettringite (C_6_AŠ_3_H_32_) (Eq. 1). If there is no Ca(OH)₂, the hydration of ye’elimite creates gibbsite and ettringite; if there is no sulfate, it will produce monosulfoaluminate and gibbsite instead. (Eq. 3). If calcium hydroxide is present, then pure monosulfate or ettringite can theoretically form, similar to the case of C4A3Š and CŠH2 (Eqs. 4, 5)^26–32^.

It is clear that the late formation of ettringite could cause the hardened cement paste to expand, which might result in large cracks. To prevent this issue, higher water/cement (w/c) ratios are needed. Greater water/cement (w/c) ratios are required to avoid this risk. El-Alfi et al.^33^ recycled marble sludge waste for preparing the Ca sulfoaluminate-belite cement in weight%: 25% kaolin, 20% gypsum & 55% marble sludge waste^33^. Hu et al.^34^ investigated the micro-mechanical properties of the CSA cement and their control by the microstructures^34^. The CSA cement in the concrete improves strength against sulfates, makes it less permeable, increases resistance to chemicals, and reduces the risk of alkali-silicate reactions. Furthermore, according to the composition of the CSA cement and its hydration outputs, rapid-hardening concrete, shrinkage-compensating concrete (SHCC), and self-stressing concrete cause the improved CSA properties to be achieved by more research studies^35–37^.

Substituting the OPC with small amounts of CSA additives could be effectively utilized to improve and control their characteristics, like the expansion or setting time that is used in the USA for type K cement (ASTM C845-04) and to manufacture shrinkage-compensated or expansive cements^38–40^. Blends of OPC with CSA cement having an elevated percentage of the CSA cement (more than 50%), and OPC hydration (especially alite, C_3_S, which is the main component of the OPC) mostly take place after numerous hydration days, forming strätlingite (C_2_ASH_8_) and portlandite (Eq. 6)^41,42^. Furthermore, when the OPC is used without any additives or mixed with low CSA ratios, alite hydration can form C-S-H gel and Portlandite, where the OPC includes C_3_A and its hydration produces ettringite (Eq. 7) and forms monosulfoaluminate in the presence of an insufficient amount of calcium sulfate. Without calcium sulfate, C3A creates unstable calcium aluminate hydrates (like C_4_AH_13_), which then turn into a stable form called hydrogarnet hydrate (C_3_AH_6_).

6 $${{\text{C}}_{\text{3}}}{\text{S}}\,+\,{\text{AH3}}\,+\,{\text{6H }} \to {\text{ }}{{\text{C}}_{\text{2}}}{\text{AS}}{{\text{H}}_{\text{8}}}\,+\,{\text{CH}}$$

7 $${{\text{C}}_{\text{3}}}{\text{A}}\,+\,{\text{CS}}\,+\,{\text{32H }} \to {\text{ }}{{\text{C}}_{\text{6}}}{\text{A}}{\check{S}_{\text{3}}}{{\text{H}}_{{\text{32}}}}$$

Many previous studies looked at how OPC and CSA cements hydrate, both on their own and when mixed together in industry. However, only a few focused on making CSA cements in the lab and then examining how replacing OPC with CSA affects the physical, mechanical, densification, and electric properties of the mixtures. Therefore, this study will investigate and discuss the effects of substituting OPC with CSA cements on physical, mechanical, densification, and electric properties. This study aims to decrease the global warming implication via exploitation, as the fractional replacement of OPC will support disbanding the ecological and economic troubles of the OPC production. Furthermore, this work concentrated on elevating the physical and mechanical characteristics of the OPC pastes by utilizing fabricated CSA clinker, which was produced by an energy-saving technique. Additionally, this study focused on improving the physical and mechanical properties of the OPC pastes by using CSA clinker made through an energy-efficient method. Other additives can support the applied workflow, enhancing the physical and mechanical properties of the OPC.

## Materials & applied techniques

### Starting Raw materials

The main starting materials used for this study are OPC (having a surface area of 3580 cm²/g), gypsum (hemihydrate, CaSO₄0.1/2(H₂O)), limestone, and bauxite. The CSA clinker mainly consists of ye’elimite (Klein’s salt, C_4_A_3_Š)^43–47^, which was fabricated in the laboratory by heating the mixture of the limestone, bauxite, and the calcium sulfate at 1250 °C. The surface area of the CSA clinker was measured using the N_2_ adsorption (BET) method, and it is 4409.3 m²/kilogram. The specific gravities of the OPC and the CSA clinker were measured using a helium pycnometer at 16 psi and room temperature; they are 3.2921 and 2.7339 g/cm^3^, respectively. The chemical compositions of the OPC, CSA, and bauxite were investigated using the XRF (Bruker S4 with an Rh source and 2.2 kW power) and are presented in Table 1, whereas the various mineralogical phases of the OPC are presented in Table 2. Many authors^48^ indicated that the stoichiometric calculations delineated the main mineral composition of the OPC.

Therefore, in the first step of the applied workflow, the CSA clinker, which primarily consists of the ye’elimite as a main hydraulic phase, is fabricated thermally in the lab at a temperature of 1250 °C. In the second step, the partial replacement of the OPC by the CSA is employed. The percentages of the CSA clinker and the OPC in the four OPC–CSA cement pastes utilized in this research are listed in Table 3.

**Table 1:** The chemical composition of the OPC, CSA clinker, & the bauxite using the XRF sequential spectrometer (WD-XRF, mass, %).

Oxides	OPC (%)	CSA (%)	Bauxite (%)	Gypsum (%)	Limestone (%)
SiO_2_	20.04	0.31	1.48	1.74	8.19
Al_2_O_3_	4.10	28.30	64.88	0.51	0.89
Fe_2_O_3_	4.20	0.05	0.09	0.27	0.48
CaO	61.31	51.50	0.26	32.46	49.11
MgO	2.46	0.40	0.25	0.54	1.21
Na_2_O	0.42	0.05	0.27	0.05	0.10
K_2_O	0.33	---	---	0.07	0.15
SO_3_	2.40	17.90	0.09	43.99	0.11
Mn_2_O_5_	0.31	---	---	---	---
ZnO	---	0.04	---	---	---
P_2_O_5_	0.18	0.03	---	---	---
TiO_2_	0.31	---	---	---	---
SrO	---	0.1	---	---	---
Cl^−^	0.06	1.19	0.05	---	0.01
LOI	3.84	1.19	32.60	20.36	39.51
Total	99.96	99.94	99.98	99.76	99.99
Insoluble residue	---	---	---	1.30	---

**Table 2:** Mineralogical phase composition of the OPC (mass, %).

Phase	OPC
Alite (C_3_S)	56.83
Belite (C_2_S)	14.68
Tri calcium aluminate (C_3_A)	3.77
Brownmillerite (C_4_AF)	12.76
Lime saturation Factor (L.S.F)	0.94

**Table 3:** Percentages of the OPC and CSA clinker proportions (in weight percentages) in the investigated mixtures.

	OPC (%)	CSA (%)
**A**	100	0
**B**	99	1
**C**	98	2
**D**	97	3

### Samples Preparation and mixing procedure

This study prepared the OPC mixtures following the European Concrete Standard (EN 206-1:2013/NA:2015)^49^ and BS EN Cement (197-1, 2011/2019-BSI)^50^. We prepared the dry cement mixtures by partially substituting the OPC with the CSA at four various percentages (0, 1, 2, and 3%). Next, the right amounts of OPC and CSA, based on the percentages shown in Table 3, were mixed together and ground for 30 min using a top planetary ball mill to create uniform mixtures.

Furthermore, we used the Vicat Apparatus^51^ to estimate the setting times and water consistency for all cement mixtures. For each constituent blend, cubic molds measuring 2.5 cm in length were utilized to mold the cement pastes, which were stored for 24 h at 100% relative humidity (RH). Thereafter, the cement mixture samples (CSA-OPC) were exposed to ordinary curing times (at room temperature under tap water) up to 90 days as a maximum period of the hydration process. For each curing hydration time interval (1, 3, 7, 28, and 90 days), the mean UCS value was determined for the 3 cubes using Ton-Industry equipment. The physical properties checked for all hydration times included apparent density (ρ_b_, g/cc), specific gravity (ρ_SP_, g/cc), and connected porosity percentage (∅, %), following the ASTM standards (C140 and C150)^52,53^.

Then, the crushed samples of the hydrated pastes were milled, and the hydration process was discontinued by stirring with a mixture of methanol and acetone for one hour^54^. Specimens were filtrated and dried for two hours at 60 °C. The samples were then kept in a tightly sealed container with CaCl₂ and soda lime for further tests (SEM, XRD, IR, & DTG/TGA).

### Synthesis of the CSA clinker

To prepare the CSA phase, the starting materials (bauxite, gypsum, and limestone) were mixed carefully together in the right amounts, ground in a ball mill for 60 min, and turned into a thick paste with a little water (about 5%), then pressed into cylindrical molds at a pressure of 20 MPa^18^. This 5% ratio was designed following the recommendation of Cao and Li^46^, who referred to the best percentage of water content to be added to the cement as 5% at maximum. Generally, producing the CSA cements consumes low energy during the manufacturing process at temperature interval 1200–1300 °C. When the raw materials were burned at 1250 °C, the required Klein’s component, which is made up of 4CaO.3AI_2_O_3_.SO_3_ (C4A3Š) and includes ye’elimite appeared clearly) as seen in XRD, Fig. 2). As there is a continuation of this study and its preparation at different temperatures. Thereafter, samples were dried at 100 °C in a hot air oven overnight and then burned at calcination temperature in an electric muffle furnace at 1250 °C with an appropriate heating rate. Finally, the cooled clinker was ground using a top planetary ball mill for 30 min, resulting in a BET specific surface area of 4409.3 m²/kg.

In the second step, the CSA clinker has a maximum amount of ye’elimite (C4A3Š) that was created at a firing temperature of 1250 °C, which is 200 °C lower than that required for the OPC clinker, resulting in lower energy use. During this step, 0.216 kg of CO_2_/kg of clinker is emitted, i.e., liberating 62.7% less CO_2_ and 33% less grinding energy in comparison to the OPC cement^21,47^.

### Characterization procedures

#### Chemical and mineral compositions

The chemical composition was performed using the WD-XRF (wavelength dispersion XRF sequential spectrometer) as presented in Table 1. Additionally, XRD analysis of the raw materials and the hardened cement specimens was performed at 30 mA and 40 KV using an XꞌPert PRO PW 3040/60 (Panalytical) diffractometer supported by a monochromatic Cu-K*a* radioactive source. Besides, FTIR spectra were registered for the dried paste mixtures and the hardened samples utilizing JASCO FT/lR-6100. The spectra were registered in the range between 400 and 4000 cm^− 1^ at 25 °C with 4 cm^− 1^ resolution.

#### Setting times & water consistency

The setting times and normal water consistency of every paste were estimated utilizing the Vicat test method according to the ASTM Designation C-191 (100% of relative humidity, RH).

#### Morphology and microstructure

The morphology and microstructure of the hardened cement mixtures were delineated by a scope emission SEM at the secondary electron mode at 15 kV as operating voltage, supported with EDX.

#### Unconfined compressive strength (UCS)

The average UCS value for three cubes that were cured for the same amount of time was estimated and recorded using programmed SHIMADZU hydraulic testing equipment at a rate of 0.025 KN/mm^2^/s with a maximum capacity of 1000 KN and prior hydration ages as mentioned in the ASTMC109^55^.

#### Density and porosity measurements

For determining the apparent density (ρ_b_ in g/cc), porosity (∅, %), and the specific gravity (ρ_g_ in g/cc) values, the cubic specimens were weighed dry (wt) utilizing an electric balance (0.1 mg accuracy), and their dimensions as well as their bulk volumes (vb) were estimated utilizing a digital caliper (0.00001 m). Then the ρ_b_ was evaluated as

8 $${{\text{\varvec{\uprho}}}_{\text{b}}}\,=\,{\text{wt}}/{\text{vb}}$$

Next, the vg was measured utilizing a helium pycnometer at 16 psi and 25 °C, and the porosity (∅) and the specific gravity (ρ_sp_) were calculated as follows.

9 $${{\text{\varvec{\uprho}}}_{{\text{sp}}}}\,=\,{\text{wt}}/{\text{vg}}$$

10 $$\emptyset ={\text{ 1}}00{\text{ }} \times {\text{ }}\left( {{\text{vb}} - {\text{vg}}} \right)/{\text{vb}}$$

To obtain reliable measurements, the porosity and density measurements were reiterated 5 times/sample, and the mean value was estimated. Table 4 displays the specific gravity, apparent density, and porosity data of the various mixtures. The measuring techniques have published by many authors^56–61^.

## Results and discussion

### Characterizing the used Raw materials

**Fig. 1:**
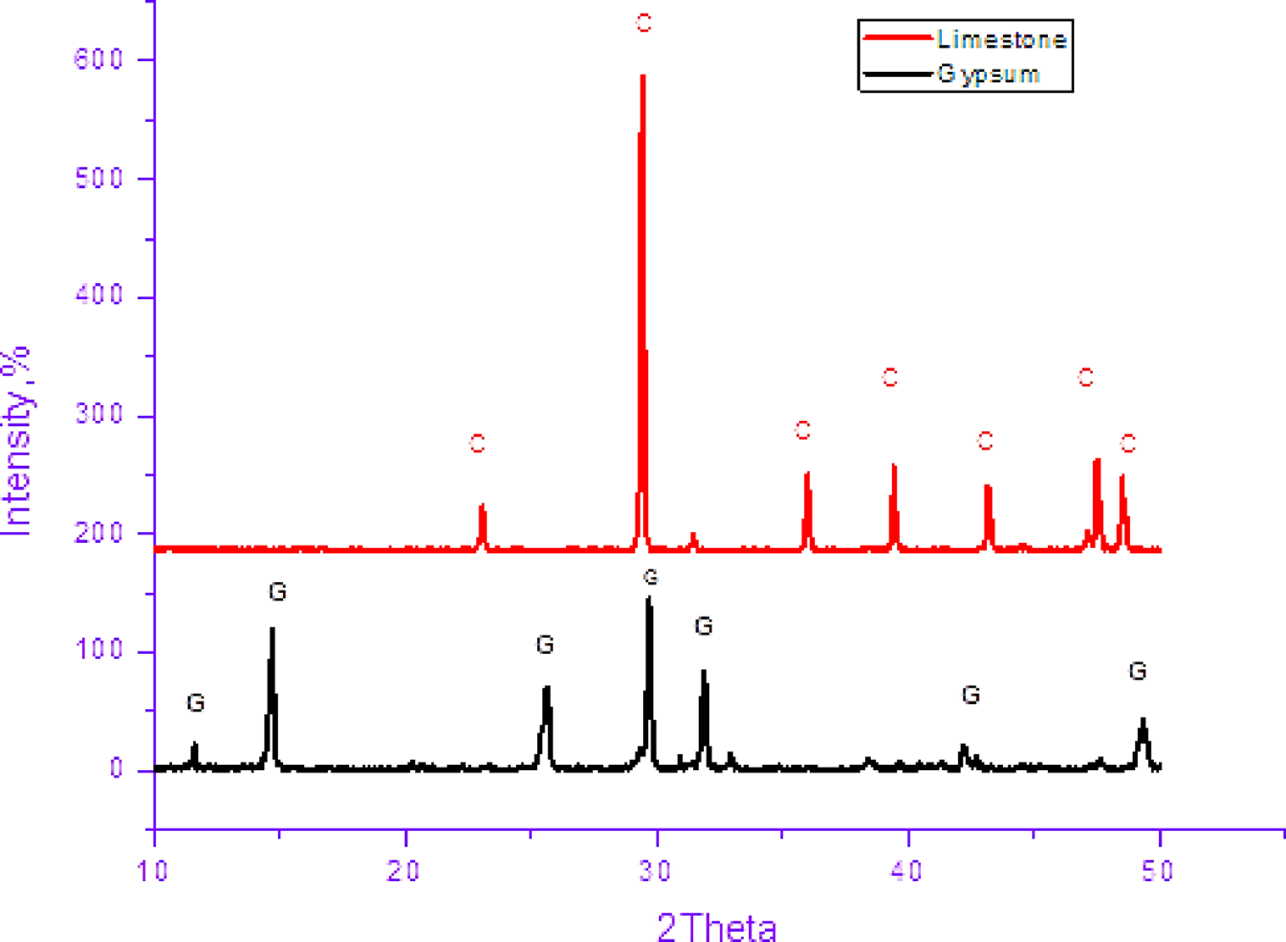
X-ray diffraction pattern for the used gypsum and limestone. C: Limestone (calcite), G: Gypsum.

The XRD analysis of the starting raw materials (Fig. 1) indicates the existence of pure crystalline hydrous calcium sulfate (gypsum, CaSO_4_.2H_2_O) and hemihydrates calcium sulfate phases (Bassanite, CaSO_4_.1/2H_2_O) assigned with a series of sharp peaks at 11.646°, 31.85°, and 33.36° with absence of any imperfection phases. The XRD pattern also displays that the used limestone is pure with no impurities, it consists of calcite mineral (CaCO_3_) with its main peak noticed at 2θ = 29.4°.

### Characterization of the prepared CSA clinker

#### XRD (X-ray diffraction)

**Fig. 2:**
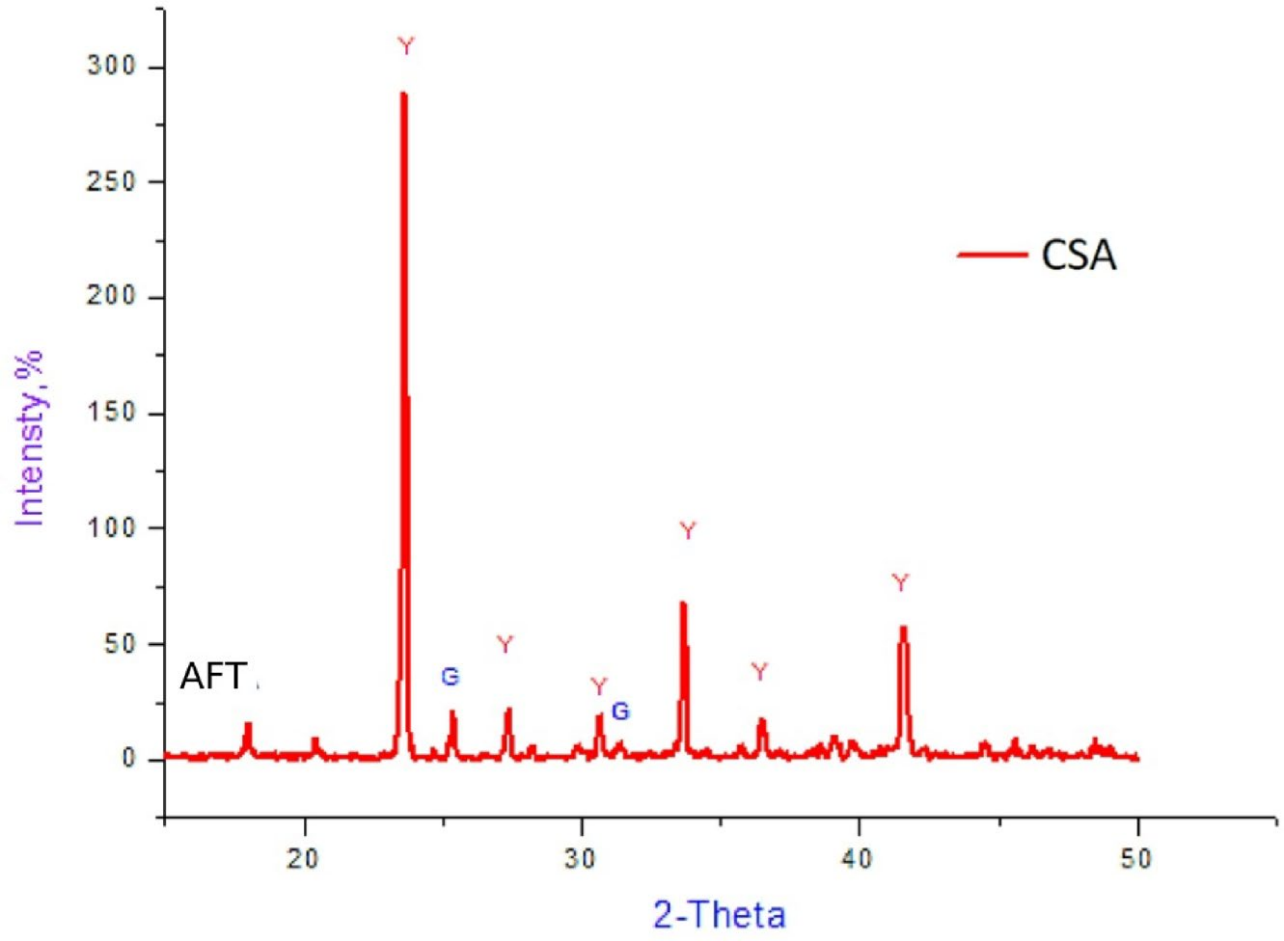
X-ray diffraction pattern for the CSA clinker. Y: Ye’elimite G: Gypsum AFT: Ettringite.

The XRD patterns of the blended CSA sample affirms the presence of the main phase of ye’elimite (C_4_A_3_Š) at diagnostic peaks 2Ɵ = 23.66°, 27.45°, 33.82°, 37.22°, and 42.19° (Fig. 2). Ye’elimite is the major phase of the clinker production fired at 1250 °C, which is less than the OPC clinker by about 200 °C, with the main peak noticed at 23.66°. The characteristic peaks of the gypsum can also be monitored but are extremely depressed, illustrating that the majority of the gypsum dissociates, producing another target phase. However, the CSA cement clinker contains minor amounts of undesirable mineral phases, such as gehlenite.

Therefore, the mineral phase fabrication of the CSA clinker that is prepared by the reaction of starting materials and the dissociation of gypsum is idealistic. These assignments are in accordance with those investigated in the published research^3,46,62–64^. Thus, the CSA clinker liberates about 62.7% less CO_2_ in comparison with the OPC cement clinker^21,46,58^.

#### TGA/DTG thermograms

**Fig. 3:**
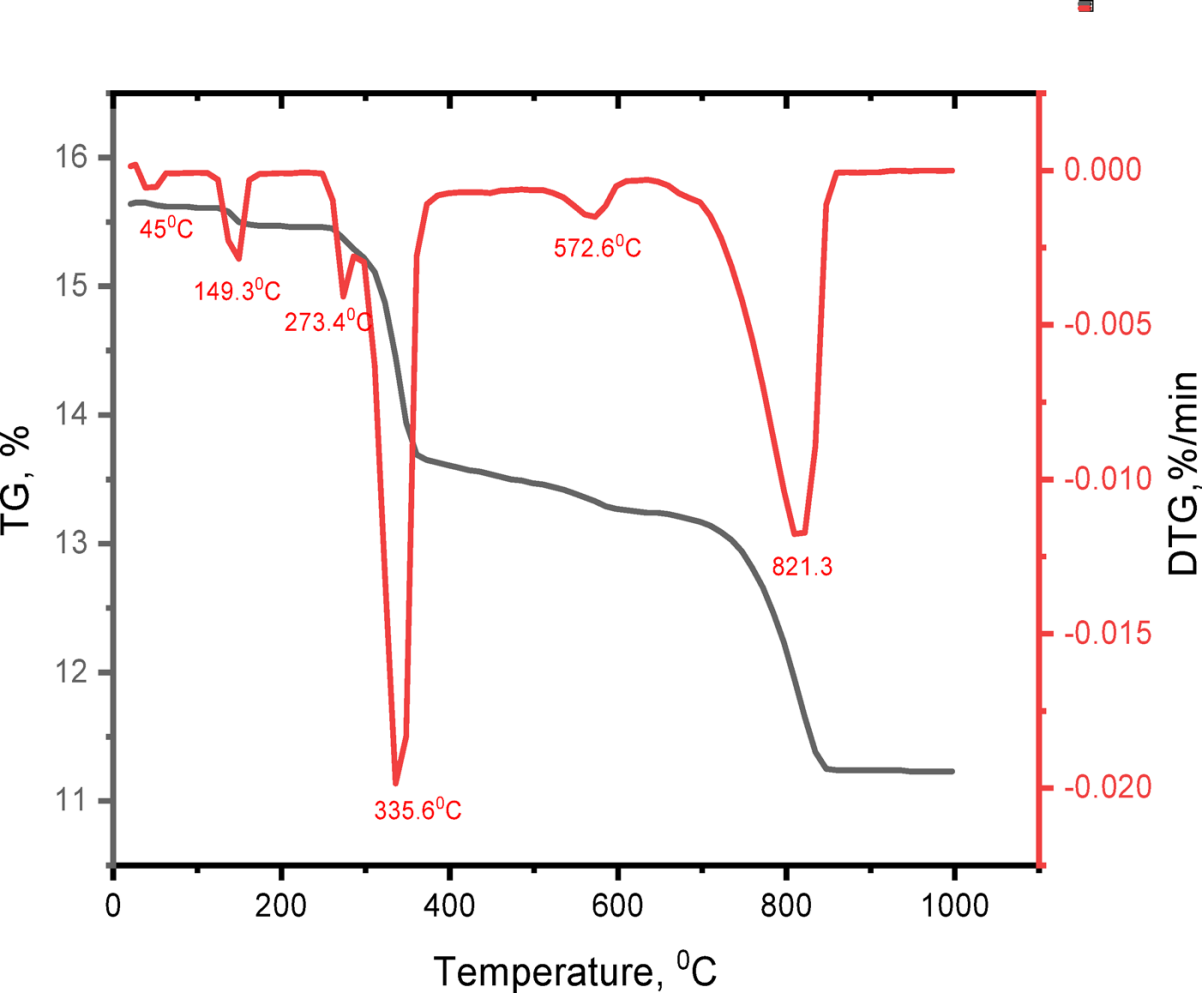
Thermogravimetry analysis curve (TGA/DTG) of the raw materials utilized for producing the CSA clinker with pure ye’elimite raw mixture.

The differential thermal analysis (DTG) and the thermogravimetry analysis (TGA) curves for the prepared CSA clinker sample clarify the appearance of six endothermic peaks presented at temperatures equal to 45.0°, 149.3°, 273.4°, 335.6°, 572.6°, and 821.3 °C, respectively (Fig. 3). The thermogravimetry analysis (TGA) data presents the temperature ranges where significant weight losses occur, while maintaining the same proportions. The first loss at 50–148 °C, with about 1.083 wt %, indicates a fractional dehydration of the gypsum phase and formation of hemihydrates. The second endotherm observed at 250–280 °C with Tmax at 271 °C confirms the entire gypsum dehydration. The third endothermic peak is in the range of 300–390 °C with Tmax at 339 °C, related to the removal of the crystallization water and the thermal decompositions of the bauxite (Fig. 3). The weight loss % for the second and third endothermic peaks was 11.786%. The fourth endothermic peak monitored in the range of 545–625 °C with Tmax at 575 °C expressed the destruction of the MgCO_3_, with about 1.045% weight loss. The fifth endothermic peak, situated at about 700–890 °C with Tmax at 824 °C, is attributed to the dissociation of the CaCO_3_ and the mass reduction % for this peak is 12.77% weight loss^65,66^. As stated in Fig. 3, the total weight loss of raw ingredients reached 28.19% during the heating process.

### Physical and mechanical properties

#### Setting times & water consistency

**Fig. 4:**
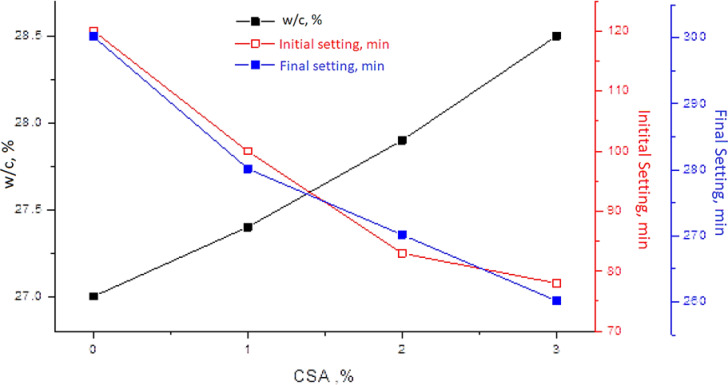
Effect of adding the CSA (1, 2, and 3%) on the water consistency and the initial and final setting times of the OPC.

Figure 4 illustrates the impact of adding the CSA on the setting times and the water consistency of the OPC are shown in Fig. 4. In general, it is expected that the prevalence area of the fresh cement mixtures and the CSA pastes reflects their workability, which Increases with increasing the water content^67^. Also, the workabiIity of the fresh cement pastes probably resulted from the high surface area of the CSA clinker (4409.3 m^2^/g), which requires a relatively high water content to attain the optimum workability. Hence, as the OPC replacement ratio increases, the water-to-cement (W/C) ratio of the OPC-CSA pastes increases due to the formulation of C_4_A_3_Š as an indispensable constituent in the CSA clinker and the need for more water to hydrate and form the primary crystallized ettringite (AFt) with a large water combination^68,69^. The results stated that the initial and final setting times have been shortened by the increase in the CSA ratios in the cement pastes, which could be due to the ettringite needles formation as a major hydration product of the C_4_A_3_Š in the CSA clinker^70^. Evidently, to comply with the European Standard EN 206-1, reducing the initial and final setting times of the OPC-CSA pastes needs adding 1–3 wt% of the CSA to the OPC. The improvement was fundamentally attributed to the development of more calcium sulfoaluminate hydrates as AFt and also the relatively high surface area of the CSA clinker, which shortened the setting times.

#### Unconfined compressive strength (UCS)

**Fig. 5:**
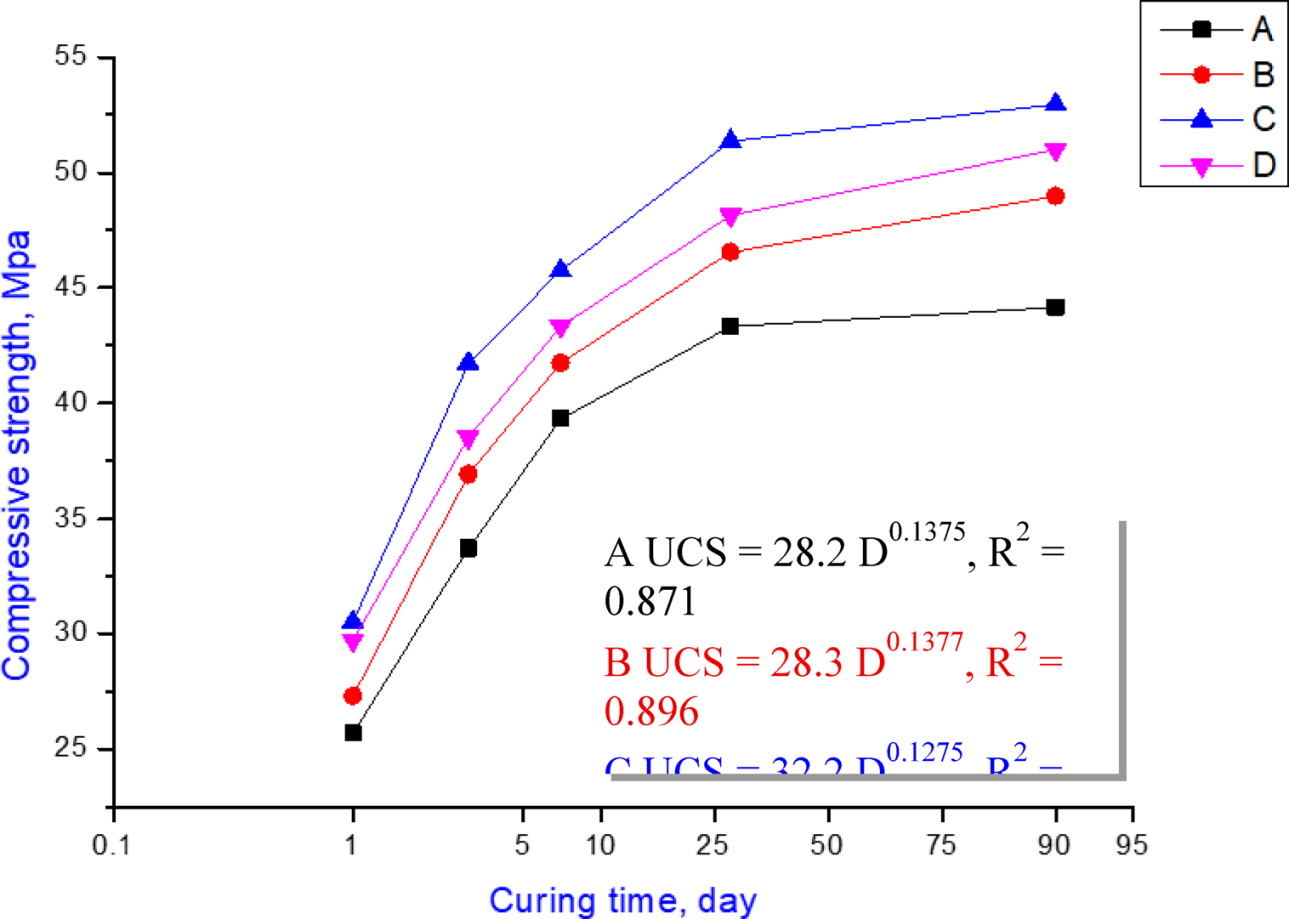
Compressive strength values of the prepared samples, OPC (sample A), OPC-1CSA (sample B), OPC-2CSA (sample C), and the OPC-3CSA (sample D).

Figure 5 reveals the unconfined compressive strength values as excellent indicators for improving the mechanical characters of the hydrated OPC-CSA cement pastes after various hydration times up to 90 days. This figure indicates that all hardened pastes (OPC, OPC-1-CSA, OPC-2-CSA, and OPC-3-CSA) consistently exhibit a persistent increase in UCS values over various hydration times ranging from 1 to 90 days. This improvement is attributed to the hydration development of the OPC clinker mineral phases (C_3_S, β-C_2_S) and the formation of hydrated yields such as the calcium silicate hydrates (CSHs). Also, the strength development is because of (1) the hydration mechanisms of two polymorphs of ye’elimite (orthorhombic & cubic), (2) the generation of calcium sulfoaluminate hydrates (CSA, ettringite AFt and AFm as shown in Eq. 11), and (3) the formation of gismondine (hydrated calcium aluminosilicate) as a result of the reaction between the active belite and the amorphous AH_3_. Evidently, supporting the Ordinary Portland Cement (OPC) with calcium sulfoaluminate (CSA) positively affects the unconfined compressive strength (UCS) values; as the percentage of CSA increases from 1 to 3%, the compressive strength (CS) magnitudes improve at each hydration time point (1, 3, 7, 28, and 90 days). This improvement in the strength values was attributed to many reasons: (1) the hydration of ye’elimite phase and the formation of ettringite (AFt); (2) all the CSA cements presented very fast strength evolution potentially owing to the rapid hydration of ye’elimite, which encourages the prompt formation of ettringite^5,71^. The hydration rate of the ye’elimite is higher than that of the calcium silicates (C_3_S, β-C_2_S) especially at an early age of hydration. Accordingly, the UCS values of the OPC-CSA samples are generally more than those of the pure OPC samples; (3) as affirmed in Sect. "Starting raw materials" & "Samples preparation and mixing procedure", the surface area of CSA clinker (4409.3 m^2^/kg) possesses an indispensable impact on the attitude, including the fast hydration reaction leading to the formation of high quantities of the hydrated products; and (4) the suitable doses of the CSA (1–3% replacement according to the European Concrete Standard, EN 206-1:2013/NA: 2015)^49^ and the BS EN Cement (197-1, 2011/2019-BSI)^50^. As the CSA replacement increased, the UCS values were enhanced because of the production of extra amounts of new hydration yields of calcium sulfoaluminate hydrates (ettringite) and the hydrated calcium aluminosilicate besides CSHs. Furthermore, the liberated lime resulted from the OPC hydration that is needed for the ye’elimite activation, and creating additional amounts of new hydrates is attributed to intensifying the mechanical properties of the hydrated pastes.

In other words, the OPC-2CSA paste (sample C) displays the highest unconfined compressive strength magnitudes due to the formation of greater amounts of ettringite and CSHs, leading to a more intensive microstructure than the OPC-3CSA paste (sample D) due to the formation of excess quantities of hydrated calcium aluminosilicate (gismondine) besides CSHs compared to those of the other specimens (OPC-1CSA, sample B; and OPC, sample A) at most hydration ages. Particular improvement in the maximum strength occurred between 1 and 7 curing days, with 28 days being the optimum curing age.

From the economic perspective, the OPC-2CSA (sample C) and OPC-3CSA (sample D) cements are regarded as the most appropriate cements, which generate ecological and economic advantages by minimizing the quantities of mischievous gases (CO_2_ and NO_x_), where the CSA clinker liberates about 62.7% less CO_2_ compared to OPC cement clinker, preserving the environment by economizing energy resources and safeguarding huge quantities of natural resources every year. Hence, the CSA clinker, which is more friable and softer, decreases the energy demanded for grinding by about 33%^21,46,58^.

11 $${\text{4}}{{\text{C}}_{\text{4}}}{{\text{A}}_{\text{3}}}\check{S}{\text{ + 80H }} \to {\text{ }}{{\text{C}}_{\text{6}}}{\text{A}}{\check{S}_{\text{3}}}{{\text{H}}_{{\text{32}}}}+{\text{ }}{{\text{C}}_{\text{4}}}{\text{A}}\check{S}{{\text{H}}_{{\text{12}}}}\,+\,{\text{2}}{{\text{C}}_{\text{3}}}{\text{A}}{{\text{H}}_{\text{6}}}\,+\,{\text{8A}}{{\text{H}}_{\text{3}}}$$

From the energy consumption perspective, the CSA additives to reduce the required heat are considered optimal alternatives for adding coal as a source of heat during the calcination process, as reported in some literature^3,46^. However, the low CSA clinker dosage may limit its overall carbon footprint reduction. Additionally, the cost of producing CSA clinker surpasses that of OPC by a significant margin.

#### Porosity (Ø) and apparent density (ρ_b_)

**Fig. 6:**
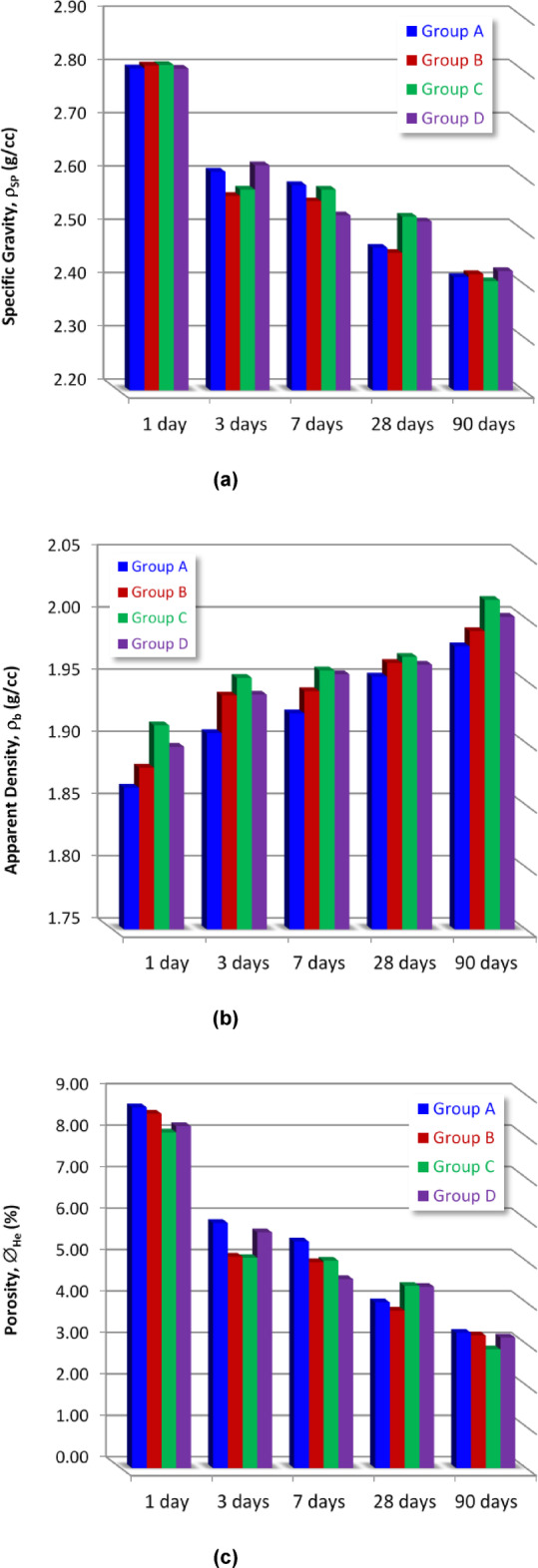
Histograms showing the change of (**a**) specific gravity, (**b**) apparent density, and (**c**) the helium porosity of the prepared four mixtures: OPC (Group A), OPC-1CSA (Group B), OPC-2CSA (Group C), and OPC-3CSA (Group D) at the various curing times (1 to 90 days).

Figure 6a displays the specific gravity (ρ_SP_) values at the various curing times (1, 3, 28, and 90 days) for the OPC (sample A), OPC-1CSA (sample B), OPC-2CSA (sample C), and OPC-3CSA (sample D) hardened mixtures. Evidently, for all samples, the (ρ_SP_) magnitudes decrease by increasing the hydration time (1–90 days) as a result of the progress of the curing process accompanied by the aggregation of the produced hydration products in the porous matrix. Additionally, the replacement of the OPC by 1–3% of CSA results in a prominent reduction in the grain density values at all curing ages. This reduction is due to the development of the hydration process of OPC minerals and ye’elimite.

On the other side, the apparent density (ρ_b_) values at different hydration times (1, 3, 28, and 90 days) for the OPC (sample A), OPC-1CSA (sample B), OPC-2CSA (sample C), and OPC-3CSA (sample D) hardened pastes are presented in Fig. 6b. The opposite trend was observed, showing that for all samples, the apparent density (ρ_b_) values increased over time (from 1 to 90 days), and the gradual replacement of OPC with CSA up to 2% led to a significant rise in the (ρ_b_) values during different curing periods, followed by a slight drop in values for sample D (3% CSA), likely due to the dilution effect of the OPC. This increase in the apparent density values was attributed to two reasons: the first main reason is the hydration of the ye’elimite phase and the formation of the ettringite (AFt), which is responsible for the early strength gaining of the cement pastes^72^. The second reason, which is considered subsidiary, is that this enhancement decreased the hydration of the clinker mineralogical phases (OPC) within the pastes owing to the early hydration process of the alite (C_3_S) and the formation of calcium silicate hydrate (CSH) gel, whilst the C_2_S hydration governs the later structure of the C-S-H group^73–76^. These reasons led to the promotion of the hydration process and the generation of extra amounts of hydration yields like CSHs, AFt, and gismondine (CaAl_2_Si_2_O_8_·4(H_2_O)) which precipitate inside the available pore spaces of the cement matrix to get a denser structure with higher apparent density. Evidently, Fig. 6b shows that the hydrated paste containing OPC-2CSA (Group C) has the highest (ρb) values at all hydration periods compared to the other mixtures.

On the other side, the porosity percentages (Ø, %) at various hydration times (1, 3, 28, and 90 days) for the various sample groups are presented in Fig. 6c. Apparently, for all specimens, the Ø decreases by increasing the hydration time up to 90 days.

**Fig. 7:**
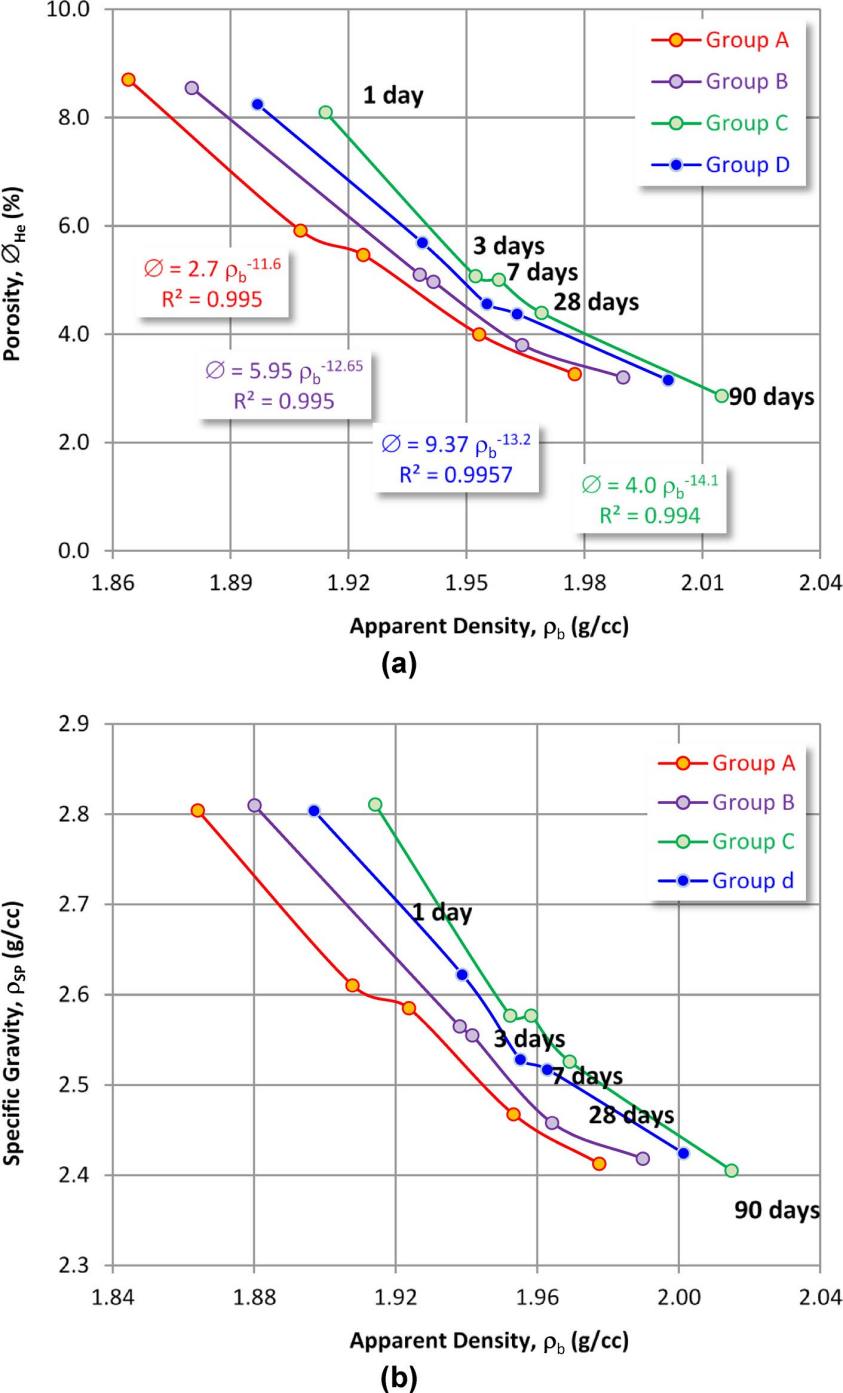
Plotting (**a**) the porosity versus the apparent density, and (**b**) the specific gravity versus the apparent density of the various sample groups at the different curing times.

Thus, the inverse relationship between density and porosity for the various batches (Fig. 7a) means that increasing hydration time increases apparent density but decreases porosity. The inverse proportional relationship between the apparent and specific density values (Fig. 7b) corroborates this statement. This decrease is important for how different hydration products are created and collected in the tiny pores, as the filling materials take up space, which significantly lowers the porosity of the hardened pastes. So, this graph shows that as the amount of OPC is gradually replaced by CSA, the percentage of porosity decreases significantly at all curing ages, which is likely due to the same hydration products mentioned earlier. In the existence of a high water-cement ratio (w/c), the hydration of CSA cement is supplied with more pore spaces for ettringite formations, preventing the self-dehydration and the spacious manner post-hardening^26,46,77^. Furthermore, producing the ettringite from the CSA cements is due to hydration without lime or elevated quantities of added calcium sulfate owing to the stabilized ettringite^78^. Therefore, it is said that increasing the amount of CSA up to 3% in place of OPC leads to a significant drop in porosity, going from 3.27% for the OPC (sample A) to 2.86% for OPC-2CSA (sample B) and 3.15% for OPC-3CSA (sample C) after 90 days of hydration. As a result of the very rapid reaction rate of ye’elimite, extra quantities of hydration yields are formed, leading to a denser microstructure of cement paste at early (1, 3, and 7 days) and later curing times (28 days). The sample OPC-2CSA (sample C) shows a notable reduction in porosity percentage.

### Phase composition

#### XRD (X-ray diffraction)

**Fig. 8:**
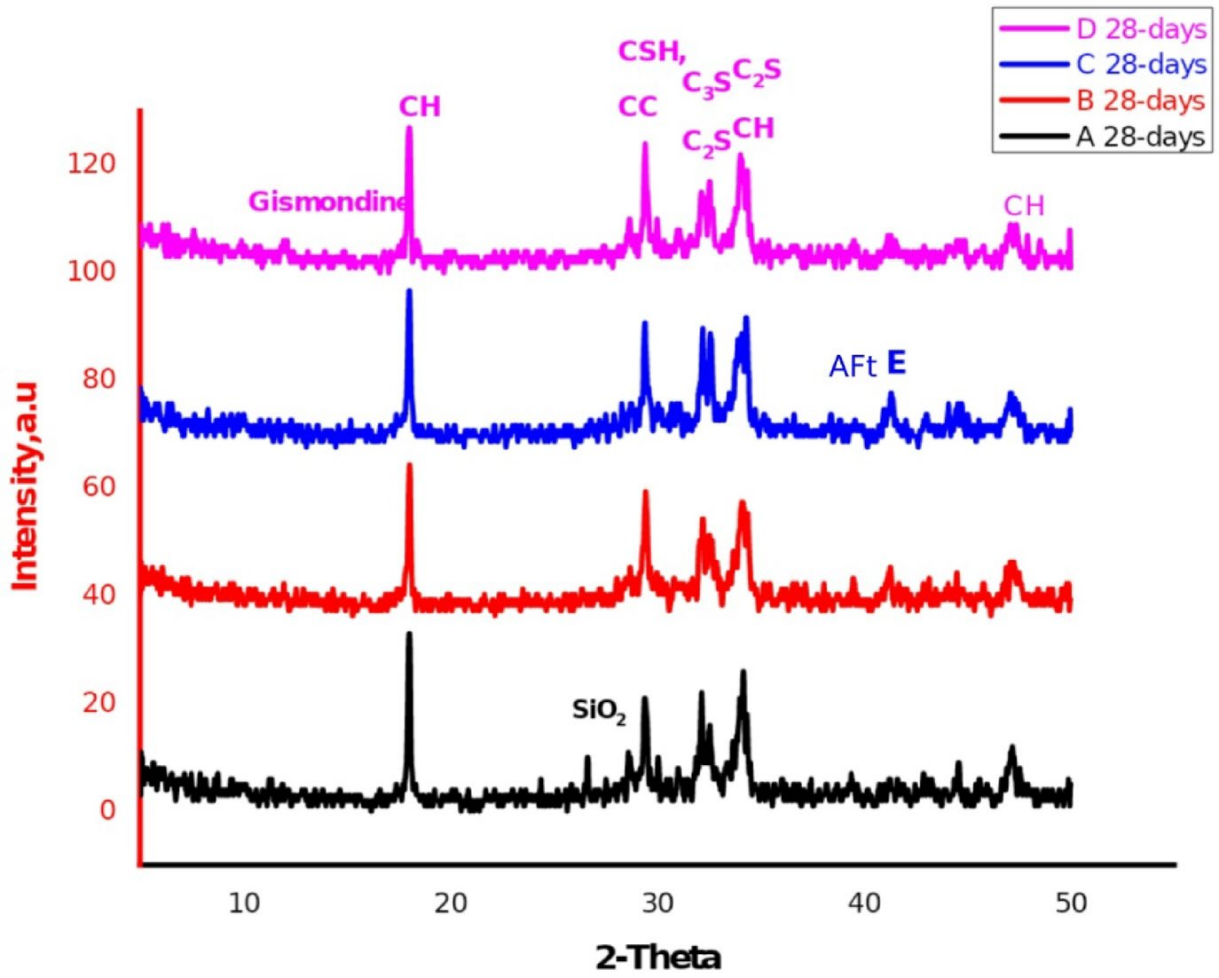
XRD patterns of the prepared samples of the OPC (sample A), OPC-1CSA (sample B), OPC-2CSA (sample C), and the OPC-3CSA (sample D) at 28 days of hydration. CH: calcium hydrate CSH: Calcium silicate hydrate C_2_S: Belite Aft: Ettringite SiO_2_: Silica.

The XRD patterns of the OPC (sample A), OPC-1CSA (sample B), OPC-2CSA (sample C), and OPC-3CSA (sample D) pastes at 28 days are presented in Fig. 8. Clearly, there are many characteristic peaks for the produced hydration yields of the OPC sample, such as CSH, CH, and some unreacted binders of C_3_S (3CaO.SiO_2_) and β-C_2_S (2CaO.SiO_2_), in addition to a distinctive peak of the calcite phase (CaCO_3_) owing to the reaction of the liberated lime with CO_2_^79–81^ as well as the appearance of the quartz phase (crystalline silica). For the OPC-1CSA (sample B), OPC-2CSA (sample C), and OPC-3CSA (sample D) pastes, it was observed that the beforehand peaks that are diagnostic for CSH, CH, βC_2_S, C_3_S, and CaCO_3_ are present aside from the characterizing peaks of new phases such as ettringite and gismondine, which were determined at 2Ɵ = 40.84° and nearly 11.06°, respectively. The appearance of the ettringite (AFt phase) during the hydration of ye’elimite (C_4_A_3_Š) is revealed by the XRD pattern with a very low intensity, proposing that the ettringite was found as very fine crystals. Thus, the ettringite can be produced by the hydration of pure CSA clinker with no need for adding more calcium sulfate (Eq. 11)^43^.

**Table 4:** Showing the specific gravity (ρ_SP_), apparent density (ρ_b_), and porosity of the prepared mixtures (samples A-D) at the various curing times.

S.No.	Curing time	ρ_b_ g/cc	ρ_SP_ g/cc	St. Dv.	∅ %
**A**	1	1.864	2.804	0.0288	8.70
	3	1.908	2.610	0.0343	5.91
	7	1.924	2.585	0.0601	5.47
	28	1.953	2.468	0.0498	4.00
	90	1.978	2.413	0.0429	3.27
**B**	1	1.880	2.810	0.0229	8.55
	3	1.938	2.565	0.0139	5.10
	7	1.942	2.555	0.0381	4.97
	28	1.964	2.458	0.0863	3.80
	90	1.990	2.418	0.0841	3.20
**C**	1	1.914	2.811	0.0319	8.10
	3	1.952	2.577	0.0194	5.07
	7	1.958	2.577	0.0143	5.01
	28	1.969	2.526	0.1389	4.40
	90	2.015	2.405	0.1073	2.86
**D**	1	1.897	2.804	0.1079	8.25
	3	1.939	2.622	0.0856	5.69
	7	1.955	2.528	0.0362	4.56
	28	1.963	2.517	0.0469	4.37
	90	2.001	2.424	0.0896	3.15

**Fig. 9:**
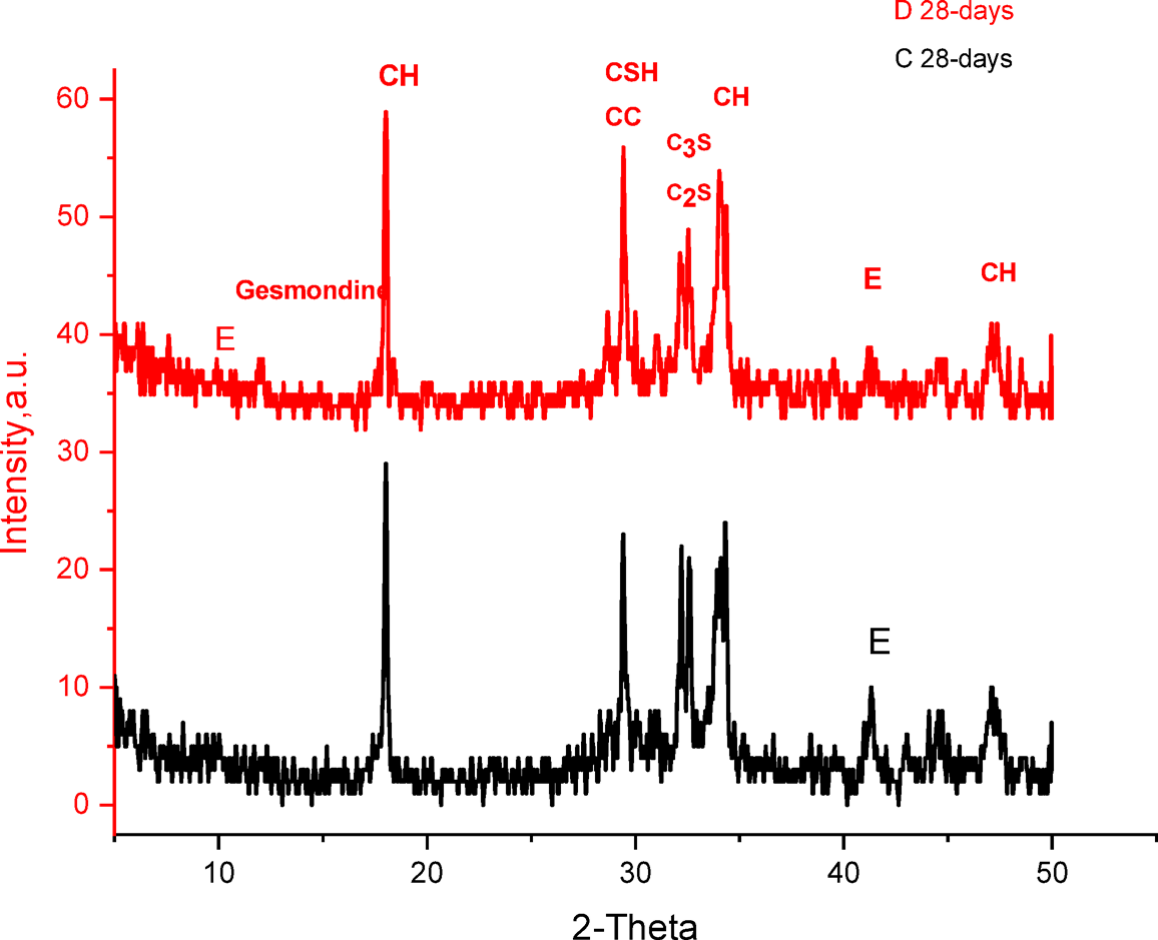
XRD patterns of the prepared samples of the OPC-2SA (sample C), and OPC-3SA (sample D) at 28 days of hydration. CH: Calcium hydrate CSH: Calcium silicate hydrate C_2_S: Belite AFt: Ettringite SiO_2_: Silica.

Furthermore, the XRD patterns of the OPC-2CSA (sample C) and OPC-3CSA (sample D) affirm the formulation of a new phase, namely gismondine (C_4_Al_**8**_Si_8_O_32_∙16 H) (Fig. 9)^82,83^, due to the reaction of the highly reactive belite, as a source of silica, with the amorphous AH3 (as indicated in Eqs. 6–7), and the portlandite attributed to the decrease in the CH and βC_2_S intensity^84^.

Evidently, the appearance of active belite restrains the production of ettringite (AFt) and monosulfatehydrate (AFm). The formation of these phases is due to the reaction of ye’elimite (C₄A₃Š) with water, which produces ettringite (AFt)^1,85,86^ and gismondine, highlighting the strong reactivity of the CSA clinker that helps create more hydration products, enhancing the physical and mechanical properties. Generally, increasing the percentage of the CSA clinker enhances the mechanical properties of all samples, particularly the OPC-2CSA (sample C), because it leads to additional hydration products that further improve these mechanical properties.

12 $${{\text{C}}_{\text{2}}}{\text{S}}\,+\,{\text{A}}{{\text{H}}_{\text{3}}}\,+\,{\text{5H }} \to {\text{ }}{{\text{C}}_{\text{2}}}{\text{AS}}{{\text{H}}_{\text{8}}}$$

#### Thermogravimetric analysis (TGA/DTG)

**Fig. 10:**
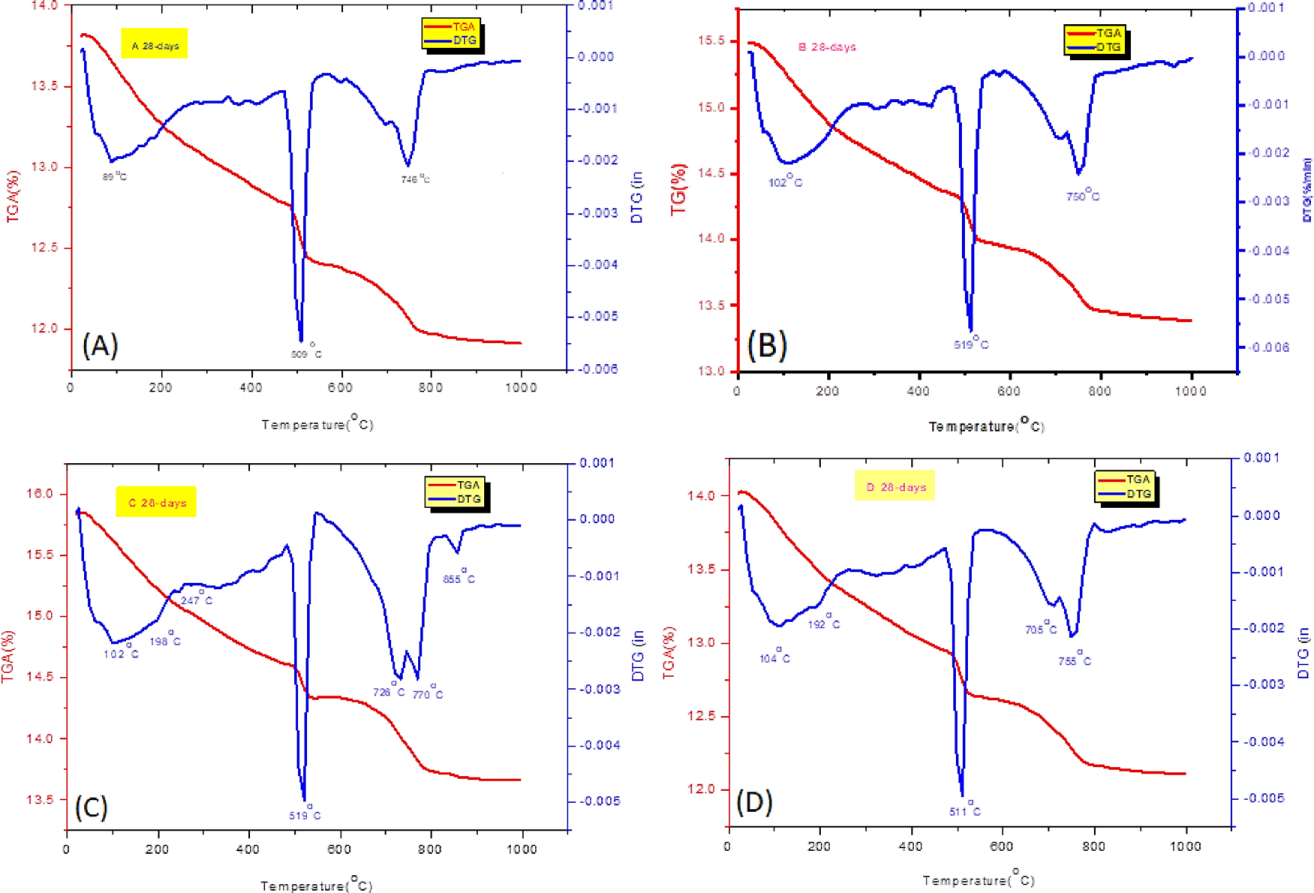
TGA/DTG thermograms of hardened specimens made from OPC (Sample **A**), OPC-1CSA (Sample **B**), OPC-2CSA (Sample **C**), and OPC-3CSA (Sample **D**) at 28 days of hydration.

Plotting the TGA/DTG thermograms data for the various hardened mixtures at 28 days of hydration in Fig. 10 indicates the presence of endothermic peaks noticed at 86–245, 240–258°, 465–538°, 614–789°, and 830–878 °C temperature ranges. The first endotherm revealed at 86–245 °C can be assigned to removing the adsorbed water molecules from the CSHs gel, the monosulfate (AFm), and/or the ettringite (AFt)^1,47,69,87,88^. Furthermore, the two endothermic peaks are recorded for the AFm^89^ in the same temperature range of 130 to 153 °C for the OPC-3CSA (sample D) hardened specimens and for the AFm at temperature 198 °C for the OPC-2CSA (sample C), respectively. These temperature ranges are ascribed to the advance of the hydration process for the cementitious particles and the formation of excess amounts of AFt, AFm, and CSHs, and the generation of more quantities of hydration products, e.g., gismondine. This methodology is in accordance with that mentioned by Scudek et al.^90^. This secondary hydration product leads to enhancing the mechanical properties of sample D. It is clearly evident that the TG analysis is helpful for the quantitative determination of ettringite, presuming that 24 water moles were lost by heating 1 mol of ettringite in the incommodious temperature range according to the strong endothermic impact^91^. The mass loss percentages of this peak after 28 days of curing were 5.6, 5.0, 5.6, and 5.4%, respectively. So, the OPC-2CSA (sample C) represented the highest mechanical characteristics. The 2nd endothermic peak was monitored at 240–258 °C with a maximum at 247 °C that confirms the dehydration of the AH_3_. Absence of the AFm and the AH_3_ in the XRD patterns may be due to their low content and poor crystallization grade. According to the hydration reaction (Eqs. 2, 3, and 11), the AH_3_ could be further hydrated and consumed, forming gismondine for the OPC-3CSA (sample D) hardened sample (Eq. 12)^89^. The mass reduction % of this peak up to 420 °C for all mixtures is 1.5, 2, 2 and 2.1% respectively. It is doubtless that percentages of the mass reducing of this endotherm are higher when gotten together with the analogue endotherm of the control OPC sample (A). This enhancement is attributed to the presence of an extra amount of hydration products of CSA cement in those mixtures (Eq. 11).

The 3rd endothermic peak detected at the temperature interval of 465–538 °C is due to the thermal dehydration of CH^47^. The percentage of weight loss for this endotherm after 28 days for all cured mixtures (samples A-D) equals 3.4, 3.3, 2.1, and 2.8%, respectively. It is clarified from Fig. 10 that the OPC-2CSA (sample C) shows the least CH value. This was attributed to the reaction between CH and the ye’elimite (C_4_A_3_Š) forming ettringite. This depression in mass reduction is assigned to the advance of the hydration reaction, and creating enormous quantities of hydration yields to improve the hardening mode. Similar results can also be confirmed by the XRD patterns (Fig. 9). At the temperature range equal to 614–789 °C, the 4th endothermic peak was determined owing to the thermal decomposition of the carbonated hydration yields produced through the store treatment of the hardened samples^91^. The three endotherms located at 720, 770, and 855 °C demonstrate the thermal destruction of the MgCO_3_ as well as the decomposition of the calcite phase, displaying diverse percentages of crystallinity. Furthermore, the percentages of the weight loss for this endotherm are 3.3, 3.3, 4.2, and 3.4%, respectively. Finally, it was shown that the total weight loss % of the various hydration products (CSHs, AFt, AFm, AH_3_, CH, and calcite) were 13.8, 13.6, 13.9, and 13. % for the studied samples (A-D), respectively, and this percentage increased to 13.853% after 28 days for the hardened OPC-2CSA (sample C). This elevation in mass loss % is associated with the hydration reaction progress and forming extra amounts of hydration products, viz., CSHs, AFm, Aft, and gismondine.

#### Transform infrared spectroscopy (FTIR)

**Fig. 11:**
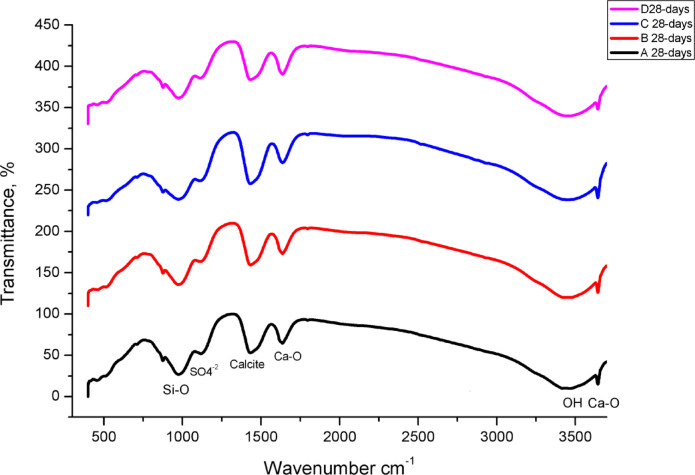
The FTIR spectra of hardened specimens made from OPC (Sample A), OPC-1CSA (Sample B), OPC-2CSA (Sample C) and OPC-3CSA (Sample D) at 28 days of hydration.

The FTIR spectra for hardened pastes after 28 days of hydration are used to highlight the chemistry of cement hydration, where the sample absorbs certain light at specific wavelengths that are unique to its chemical makeup (Fig. 11). Plotting the infrared spectra (FTIR) of the OPC hydrated sample in comparison with the OPC-CSA hydrated samples clarifies many characteristic bands for the various generated hydration yields, such as the broad feature produced at positions 3435 and 1640 cm^− 1^ (Fig. 11). This broad feature could be ascribed to the bending vibration of irregularly bound water of the CSH, ettringite, gismondine, and monosulfate hydrate^33,92,93^. The broadband may indicate a significant enhancement of the hydrated products percentage associated with water. The bands at 3648 and 1640 cm^− 1^ are associated with the inner hydroxyl group in Portlandite Ca(OH)_2_, which is created during the hydration process of the silicate phases (C_3_S and βC_2_S) producing calcium silicate hydrate (CSH). Obviously, the intensity of the Ca(OH)_2_ bands is reduced with the addition of the CSA due to the interaction of the portlandite with the ye’elimite (C_4_A_3_Š), forming ettringite.

The sulfate absorption bands are presented at 1150 cm^− 1^^94^, where its intensity decreases with the extension of the CSA as a result of the reaction with ye’elimite (C_4_A_3_Š), forming ettringite, which reinforced the mechanical properties and strengthened the structure. The asymmetric stretching Si-O band, which is located at 968 cm^− 1^, affirms the presence of calcium silicate hydrate (CSH)^95^. These dip-hump characteristics could represent depression of the belite phase and fashioning the calcium silicate hydrate (CSH) and the gismondine. The carbonate bands at 1438 and 870 cm^− 1^ are detected owing to the reactions of the atmospheric CO_2_ with the Portlandite Ca(OH)_2_^96^.

#### Scanning electron microscopy (SEM) and EDS

**Fig. 12:**
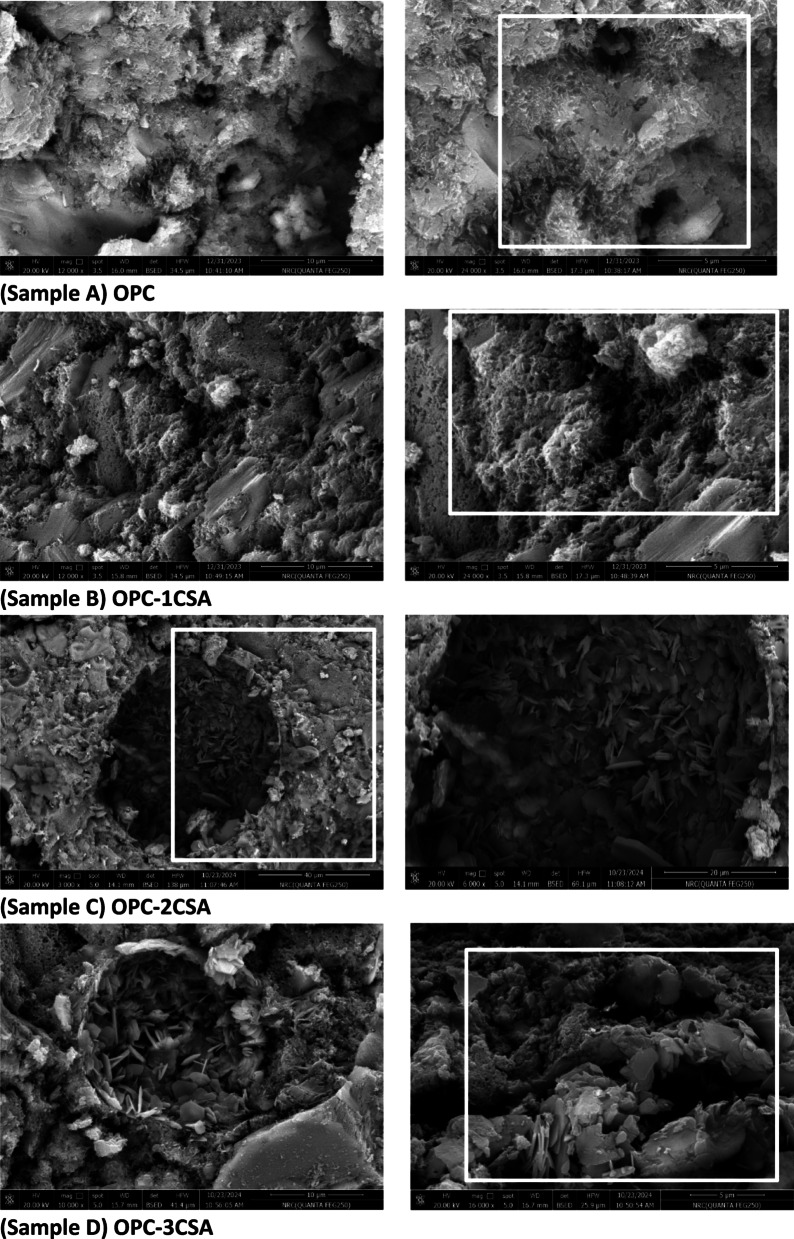
SEM micro images of hardened specimens made from OPC (Sample **A**), OPC-1CSA (Sample **B**), OPC-2CSA (Sample **C**) and, OPC-3CSA (Sample **D**) at 28 days of hydration.

To delineate the impact of adding the CSA phase on the microstructure of the hardened OPC cement paste, and the alterations in the hydration yields micromorphology of these hydrated pastes, samples were monitored using the SEM coupled with EDS (Fig. 12A-D). The internal structure, textural, and morphological features for the hardened samples after 28 days of hydration are investigated. The OPC (sample A) sample confirms the presence of small amounts of CSHs with various morphological natures, sponge & rod-like crystals, interfered with free lime hexagonal crystals aside from small quantities of calcite phase and many connected pores that are not yet prepared for the aggregation of extra hydration products (Fig. 12A). Also, the morphological characteristics of the hardened specimens made from mixing the OPC with the CSA are clarified in Fig. 12 (B-D), and the presence of different hydration productsquickened by the nucleation of calcium carbonate, as corresponding micro rods crystals and plates of CSHs, and fibres or needle like of AFt, are also stated^64^. SEM imaging clarifies that the remained β-C_2_S could react with the amorphous AH_3_ forming gismondine which already is affirmed from the XRD results. Furthermore, all these yields are shaped and intermingled with a little amount of the CH hexagonal crystals owing to the essential and additional hydration products promotion accompanied withfilling the micro and nano pores improving the physical and mechanical properties. It is generally accepted that the ye’elimite phase (C_4_A_3_Š) would be entirely hydrated by 28 days of hydration which is in harmony with the previous XRD and TGA-DTG results where, the ye’elimite phase hydrates forming ettringite phase as well as a few monosulfate C_3_A.CŠ.12H_2_O content, while the belite (βC_2_S) phase hydrates to produce the calcium silicate hydrate (CSH) and the gismondine (C_4_AL_8_Si_8_O_32_.16 H). Obviously, thenew crystals phase could be transformed into crystalline aggregates precipitate in the interstitial pore spaces of the measured specimens, resulting in a denser structure of the OPC-2CSA and OPC-3CSA (samples C& D).

**Fig. 13:**
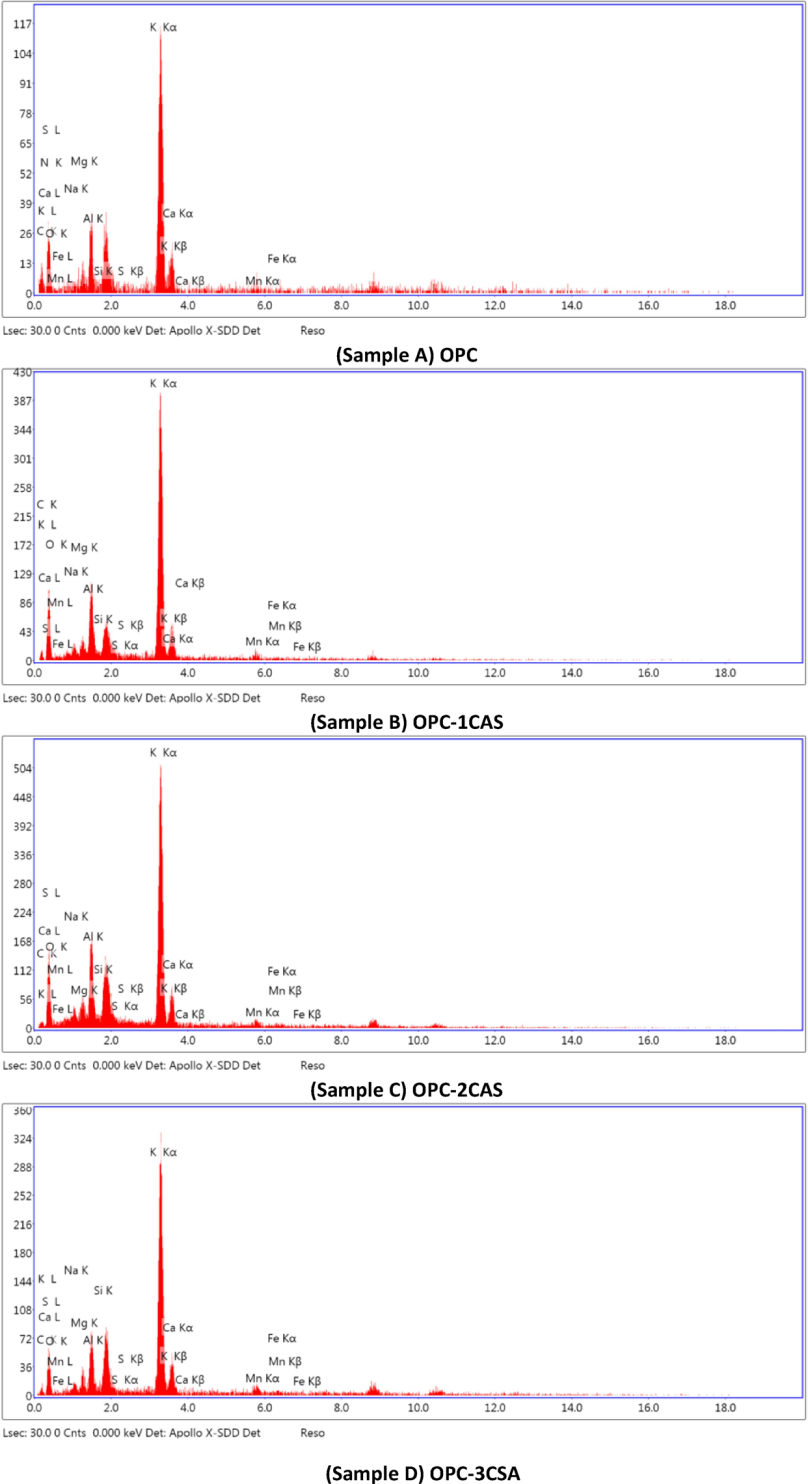
EDS patterns derived from area scanning (as shown in Fig. 12) at 28 days of hydration for hardened specimens made from OPC (sample **A**), OPC-1CSA (sample **B**), OPC-2CSA (sample **C**), and OPC-3CSA (sample **D**) pastes.

Finally, the EDX image of the hardened OPC (A) paste, after 28 days of curing time, is presented in Fig. 13A. The figure shows various percentages of silicon, sulfur, calcium, oxygen, aluminium, magnesium, and sodium explaining the likelihood of generating some phases like the CSHs, CH, and the AFt according to (Eq. 7) and leading to the expansion of the paste due to the creating of nano pores and micro crakes. On the other side, Fig. 13(B- D) shows the EDX peaks of the OPC-CSA mixtures (samples B, C, and D) after 28 days hydration time. The image emphasizes various elements (in %) that indicates the probability of formation of the same previous hydration products beside ettringite according to Eqs. (1, 2 and 11) and gismondine for Eq. (12). Therefore, adding few amounts of CSA to the OPC enhanced its physical and mechanical properties due to the hydration process and formulation of new phases, especially at 28 days as a curing age.

### The optimum CSA percentage for improving properties of the prepared cements

As a part of the ongoing search for more sustainable alternatives to Portland cements, calcium sulfoaluminate (CSA) cements have gained increased scientific attention. CSA cements are also characterized by favorable engineering properties, such as rapid setting and hardening and high early and late compressive strength, which makes them suitable for urgent repairing, sealing, and soil stabilization. The behavior of CSA cements is mainly controlled by the ettringite that has been formed upon the hydration of their main mineralogical component, ye’elimite (C_4_A_3_®S). Ettringite is characterized by an early onset of its mechanical strength, and under certain conditions, where its crystallization is associated with considerable expansion, and capable of developing shrinkage compensation in the matrix. Therefore, the kinetics of ettringite formation regulates the mechanical properties of composites containing CSA and OPC cements. Pure ye’elimite is often used as a model system for CSA^97^. The production of extra amounts of new hydration yields of calcium sulfoaluminate hydrates (ettringite) and hydrated calcium aluminosilicate besides CSHs. Additionally, the lime released from the OPC hydration, which is needed for the ye’elimite activation and creating additional amounts of new hydrates is attributed to intensifying the mechanical properties of the hydrated pastes.

In simpler terms, the OPC-2CSA paste (sample C) has the strongest unconfined compressive strength due to the formation of more amounts of ettringite and CSHs, leading to more intensive microstructure than the OPC-3CSA paste (sample D) due to the formation of excess quantities of hydrated calcium aluminosilicate (gismondine) besides CSHs compared to those of the other specimens. The classification of the gismondine as a zeolite group of hydrated aluminosilicate minerals is based on its porous structure. Gismondine can coexist with other cement phases like CSH and ettringite.

## Conclusions

In this study, the feasibility of fabrication CSA cement by sintering at a temperature of 1250 °C for the holding time (1 h) using gypsum, limestone, and bauxite was investigated. The OPC mixtures were made by replacing some of the CSA cement clinker with four different amounts (0%, 1%, 2%, and 3%) according to the European Concrete Standard and BS EN Cement guidelines (197-1, 2011/2019-BSI). The acquired mixture specimens underwent mineralogical examination using various techniques. The research’s experimental perceptions summarize the main conclusions.

1. The prepared cement sample (C) appropriates the optimum mechanical properties, after the hydration process up to 90 days, more than the other prepared cement specimens (A, B, and D). It is characterized by very low porosity (2.86%), and relatively high apparent density (2.015 g/cc), specific gravity (2.405 g/cc), and compressive strength (52.5 MPa).
2. Formation of the calcium aluminosilicate hydrate (gismondine, C_4_Al_8_Si_8_O_32_∙16 H) as a result of the reaction of active β-C_2_S or CSH with the released AH_3_ during the hydration ye’elimite phase, leading to suppression of the production of crystalline calcium sulfoaluminate hydrates in the forms of AFt or AFm as detected from the results of XRD and TGA.

## Data Availability

Data will be available on reasonable request by contacting the corresponding author: Bassem S Nabawy; bsnabawy@yahoo.co.uk; bs.nabawy@nrc.sci.eg.
